# Transcriptome Profile of Next Generation Sequence Data Related to Inflammation on Nasopharyngeal Carcinoma Cases in Indonesia

**DOI:** 10.31557/APJCP.2020.21.9.2763

**Published:** 2020-09

**Authors:** Digdo Sudigyo, Gisti Rahmawati, Dicka Wahyu Setiasari, Risky Hiskia Poluan, Salsabila Lutf Sesotyosari, Tirta Wardana, Cita Herawati, Didik Setyo Heriyanto, Sagung Rai Indrasari, Afiahayati, Indwiani Astuti, Sofa Mubarika Haryanaana

**Affiliations:** 1 *Study Program of Biotechnology, Universitas Gadjah Mada, Yogyakarta, Indonesia. *; 2 *Bioinformatics and Data Science Research Center, Bina Nusantara University, Jakarta, Indonesia. *; 3 *Faculty of Medicine, Public Health and Nursing, Universitas Gadjah Mada, Yogyakarta, Indonesia.*; 4 *Department of Computer Science and Electronics, Universitas Gadjah Mada, Yogyakarta, Indonesia. *; 5 *Dharmais Cancer Hospital, Jakarta, Indonesia. *; 6 *Universitas Jenderal Soedirman, Central Java, Indonesia. *

**Keywords:** Nasopharyngeal cancer, transcriptome profile analysis, inflammation, next generation sequencing

## Abstract

**Objective:**

Transcriptomic Profile Analysis Related to Inflammation in Nasopharyngeal Carcinoma Cases.

**Methods:**

This study used 2 control samples taken using the brushing technique and 7 cancer samples with tissue biopsy. Isolate total RNA using Rneasy^®^ RNA Extraction Mini Kit. Measurement of total RNA concentration and purity using a fluorometer and nanodrop Qubit. Synthesis of cDNA library uses TruSeq® RNA Library Preparation Kit V2 and concentration is measured using qPCR. Sequencing samples using NGS Illumina NextSeq 550 platform engine. Quality control results of sequencing using FASTQC, and raw data processing using HISAT2. Differential analysis of gene expression (*DEGs*) using edgeR and pathway analysis using DAVID and PANTHER.

**Results:**

From the 25,493 genes that experienced a significant change in expression level (P <0.05) from DEG analysis there were 13 genes that play a role in the inflammatory process. Based on DAVID pathway analysis software, there are 8 genes detected based on the KEGG pathway database found in 2 pathways, namely Inflammatory Mediator Regulation of TRP Channels pathway with genes that play *HTR2A, NGF, TRPA1, PRKCG*, and *ADCY8. CXCL9, CXCL10,* and *CXCL11 *genes are found in the Toll-Like Receptor Signaling pathway. Based on PANTHER pathway analysis software, 6 genes were found, namely *CXCL10, MYLK2, COL20A1, MYH2, ACTC1*, and *ALOX15* in the Inflammation Mediated by Chemokine and Cytokine Signaling pathways. Almost all genes found from DEGs are upregulated, except the *ALOX15 *gene that is downregulated.

**Conclusion:**

There are 13 genes that play a role in the inflammatory process in Nasopharyngeal Carcinomafrom a sample of the Indonesian population. Genes *CXCL9, CXCL10, CXCL11, MYLK2, COL20A1, MYH2*, *ACTC1, HTR2A, NGF, TRPA1, PRKCG*, and *ADCY8* have been upregulated and* ALOX15* has been downregulated. These genes play a role in the Inflammation Mediated by Chemokine and Cytokine Signaling pathways, Inflammatory Mediator Regulation of TRP Channels, and Toll-Like Receptor Signaling.

## Introduction

In Indonesia, Nasopharyngeal Carcinomais ranked as a dangerous malignant tumor seen from the level of malignancy. The prevalence of this disease in Indonesia is very high at around 48,401 in the past 5 years. While Nasopharyngeal Carcinomacases that cause death that is equal to 11,204 cases (GLOBOCAN, 2018). The cause of Nasopharyngeal Carcinomais multifactorial, while the factors that cause tumorigenesis include Epstein-Barr virus (EBV), genetic susceptibility (in certain races) and carcinogens from the environment all of which play an important role in etiology, especially in endemic types (Zhou et al., 2007). Several factors that influence trigger epigenetic dysregulation in nasopharyngeal cancer, which show a change in gene expression that is typical of the carcinogenesis process (Heyn et al., 2014). Each type of cancer itself has different gene expression patterns that the transcriptome profile of Nasopharyngeal Carcinomacan later be used as a specific biomarker by looking at its epigenetic regulation (Subramanian et al., 2005; Wang et al., 2009). Next-Generation Sequencing (NGS) is a method that can generally see several expressions of a gene, transcriptomic profile, and its effect on gene expression of all genome sequences at once (Spencer et al., 2013).

Chronic inflammation can lead to diseases such as cancer. Inflammation itself is included in one of the Hallmark of Cancer so that its role is important to be studied related to the etiology of cancer, especially nasopharyngeal cancer. Inflammation that occurs in cancer can cause epigenetic changes that can cause tumorigenesis, so the inflammatory process is often linked as the main door for cancer formation. Some pro-inflammatory products influence the process of angiogenesis, proliferation, suppression of apoptosis, invasion and metastasis that are regulated by NF-kB (Nuclear Factor Kappa B) transcription factors by expressing genes to trigger the process of cancer formation. (Epstein and Wall, 2008; Zhe Ji et al., 2018).

Therefore, NGS technology is expected to create a transcriptome profile related to inflammatory process in Nasopharyngeal Carcinomacases in Indonesia through bioinformatics analysis, so that it can be used as a reference for studies, especially related to the inflammatory process that induces carcinogenesis and genes expressed as potential biomarkers in conducting diagnosis, prognosis, and choice of gene therapy in Nasopharyngeal Carcinoma patients.

## Materials and Methods

This study obtained 9 samples that met the criteria for sequencing for NGS, which consisted of 7 NPC samples and 2 control samples taken consecutively. NPC samples were taken by biopsy method on tissue of patients diagnosed with NPC conducted by ENT specialists at Dharmais cancer hospital in Jakarta, Indonesia. The control sample was taken using the brushing method in the nasopharyngeal area of a normal individual carried out by an ENT specialist at RSUP Dr. Sardjito Yogyakarta, Indonesia.

*Isolation of total RNA and Library preparation*

Isolation of total RNA of NPC samples and control using the Rneasy^®^ RNA Extraction Mini Kit was carried out according to the kit protocol (Qiagen, CA). Total RNA concentration was measured using a Qubit fluorometer (Thermo Fisher, USA) and purity using nanodrop (Bio-Rad, CA). cDNA library was synthesized using the TruSeq^®^ RNA Library Preparation Kit V2 (Illumina, CA) according to the kit protocol, and tested for quality using qPCR (Applied Biosystem, USA).

*RNA-Seq and data quality analysis sequencing*

The cDNA sequencing was performed using the Illumina NexSeq 550 Next Generation Sequencing (NGS) engine in Eijkman Institute Jakarta, Indonesia. Sequence data quality testing is performed using FASTQC version 0.11.8 to see Quality Control analysis.

*RNA-Seq data analysis*

The results of raw data sequences from NGS were carried out transcriptomic analysis using the bioinformatics approach through Linux and windows operating systems. The Process of gene mapping alignment in raw data uses HISAT2 version 2.1.0 based on the H. sapiens UCSC hg38 reference (GRCh38) genome (https://ccb.jhu.edu/software/hisat2/index.shtml). Alignment calculations are performed using HTseq-count version 0.11.1 based on Ensembl genome browser 98 in GTF file format (ftp://ftp.ensembl.org/pub/release-98/gtf/homo_sapiens). Differential analysis of gene expression (*DEGs*) is used by RStudio software (version 1.1.456) with an edgeR package (version 2.6.10) with a gene p-value ≤ 0.05 to see the level of significance. Pathway analysis using web-based software, namely DAVID version 6.8 (https://david.ncifcrf.gov/) which refers to KEGG (Kyoto Encyclopedia of Genes and Genomes) pathway and PANTHER (http://pantrherdb.org.).

## Results

*Total RNA isolation*

From the results of total RNA isolation, measurements of RNA concentration and purity of RNA were measured to see the quantity of RNA isolated after purification which would then be done library preparation. The measurement of RNA concentration is used by the qubit Assay machine and the measurement of RNA purity is used by nanodrop. Selection of 31 isolated samples including control and cancerous tissue, only 16 samples could be used for library preparation, with the measurement results in Table 1.

**Figure 1:**
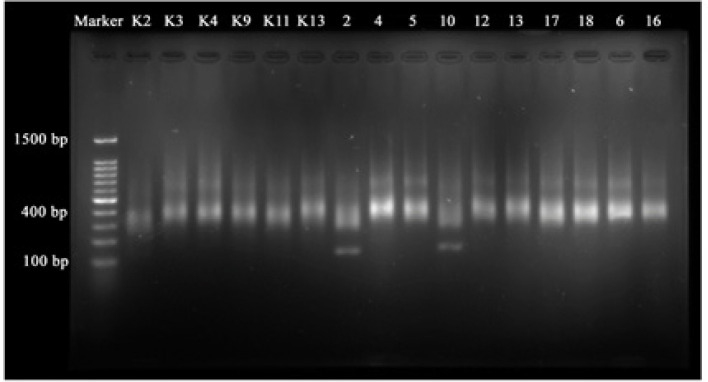
Result of Electrophoresis qPCR

The purity range of RNA with nanodrop is 1.7 - 2.1 nm (with wavelength A260 / A280). The concentration needed for the next stage using a qubit assay is at least 10 ng / µL. Based on the results of measurements of RNA quality (Table 2) a quantitive PCR (qPCR) was used to see the quality and size of fragments in basepair using electrophoresis from quality of the cDNA library, 9 samples were selected for the normalization and pooling stages consisting of 2 control samples with sample codes K2 and K4 , and 7 Nasopharyngeal Carcinomasamples with sample codes 4, 5, 6, 10, 12, 13, and 16. The cDNA library quantity obtained for the next stage is in the range of 4.73 µm to 27.61 µm (Table 2), whereas cDNA library-quality obtained fragment size of 400 bp (Figure 1).

After normalizing and pooling of the 9 selected samples, sequencing using Illumina Nextseq 550 NGS (Next Generation Sequencing) machine will be obtained to obtain the whole genome raw data in the form of fastaq format. Then quality control is performed to see the quality of the sequencing results. Based on the results of FastQC (Table 3), in 9 samples obtained fragment length with a range of 35-76 bp and value of sequence quality with poor quality of 0%. There is no sequence value with poor quality that the quality of raw data is appropriate for further bioinformatics analysis. While the value of content GC does not really affect, in the RNA-seq distribution of GC content may be larger or smaller than the range of theoretical GC content between transcripts (Andrews, 2010).

*DEGs (Differential Expressed Genes) Analysis*

From the results of edgeR, 60,617 genes were annotated with the ENSEMBL symbol database, then normalization and filtering were carried out which at the same time counted significantly and looked at the bias of the gene values expressed in each sample and obtained 25,943 genes from the calculation.

**Figure 2:**
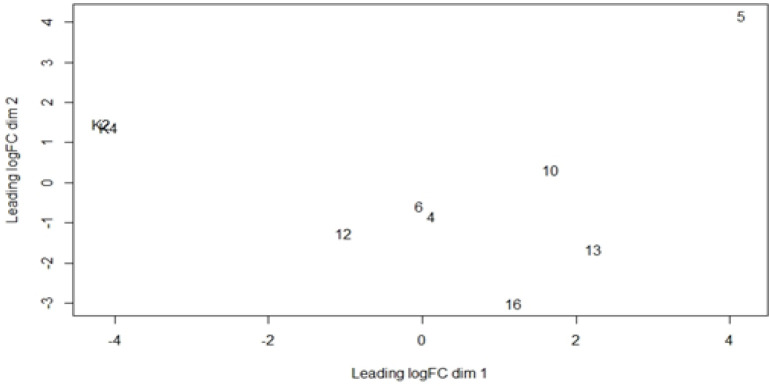
Plot MDS (Multidimensional Scaling) Using edgeR

There are 2 types of plots from DEGs analysis using edgeR, namely MDS (Multidimensional scaling) plots, and BVC (Biological Coefficient of Variation) plots. The MDS plot results (Figure 2) show that K2 and K4 have a high level of data similarity as control compared to other samples (Nasopharyngeal Carcinomasamples), and the difference between the control sample and the cancer sample is highly visible based on class analysis. The data shows that the two control samples used do not show the absence or lack of data bias and have the same level of similarity in data expression so that it can be used as a good control in DEGs data analysis (McCarthy et al., 2012).

**Figure 3:**
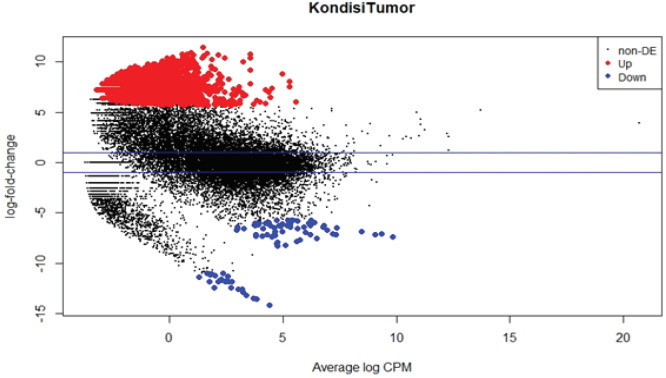
Plot BVC (Biological Coefficient of Variation) Using edgeR

The BVC plot results show (Figure 3) showing that there were 1,956 upregulated genes that were shown in red plots and 90 genes downregulated which were shown in blue plots, while 23,897 genes did not change expression levels or were called not signature. Then the DEGs data results that are known to the type of genes and level of expression are tabulated in output in the form of CSV format found as many as 2,046 genes that are significantly seen based on p-values less than 0.05.

*Pathway analysis*

In this process used DAVID and PANTHER which are web-based software by uploading genes from the results of DEGs. Based on the analysis of the pathway, both of them show different results. From the results of PANTHER analysis in (Supplementary 1), it shows the results of 337 pathways in the database only 1 was detected which plays an important role related to inflammation in Nasopharyngeal Carcinomathat plays a role in Inflammation Mediated by Chemokine and Cytokine Signaling Pathway. In the inflammatory pathway mediated by chemokine and cytokine signaling, there were 6 genes from the results of DEGs involved in this process (Supplementary 3), namely *CXCL10, ALOX15, MYLK2, COL20A1, MYH2, *and *ACTC1*.

Based on DEGs data (Table 4) of genes related to inflammatory pathways mediated by chemokine and cytokine signaling almost all showed upregulation in all genes except* ALOX15* genes that showed downregulation with fold change values of -7,165, while the highest upregulated in COL20A1 amounting to 9,403.

Based on DEGs data (Table 4)of genes related to inflammatory mediator regulation pathways in TRP channels and Toll-like signaling pathways, almost all showed upregulation in all genes with the highest fold change values in the *ADCY8* gene of 9.2334, and the lowest in the *CXCL *gene 10 in the amount of 5.8340.

**Table 1:** Concentration and Purity of Total RNA Isolation Results

No.	Sample	RNA Concentration (ng/µL)	RNA Purity (260/280 nm)
1	K2	42,80	1,905
2	K3	19,70	1,965
3	K4	73,00	1,877
4	K9	61,60	1,917
5	K11	59,00	1,752
6	K13	20,90	1,908
7	2	8,91	1,859
8	4	9,86	1,931
9	5	15,10	1,871
10	6	313,00	1,890
11	10	100,00	1,997
12	12	15,40	1,846
13	13	26,90	2,210
14	16	324,00	2,040
15	17	5,63	1,970
16	18	26,00	2,210

**Table 2:** Calculation and Sample Normalization Formula

Sample	RNA Concentration (ng/µL)	RNA Purity (260/280 nm)	cDNA Concentration (pM)	cDNA Fragment (bp)
K2	42,80	1,905	9.18	400
K4	73,00	1,877	5.23	400
4	9,86	1,931	4.73	400
5	15,10	1,871	11.82	400
6	313,00	1,890	12.77	400
10	100,00	1,997	6.28	400
12	15,40	1,846	9.65	400
13	26,90	2,210	14.44	400
16	324,00	2,040	27.61	400

**Table 3:** List of Inflammatory-Related DEGs in Nasopharyngeal Carcinoma Based on the Pantehrdb Database

No.	Entrez ID	Genes	Symbol	logFC	logFCPM	LR	PValue	FDR
1	5644	ENSG00000125414	MYH2	7.24064	-2.8544	10.459	0.00122	0.021
2	12548	ENSG00000169245	CXCL10	5.83406	3.69611	8.829	0.00297	0.041
3	10643	ENSG00000161905	ALOX15	-7.1647	7.31831	11.689	0.00063	0.015
4	2469	ENSG00000101203	COL20A1	9.40313	-0.1896	16.714	0.00004	0.008
5	10323	ENSG00000159251	ACTC1	9.04116	-0.6687	15.799	0.00007	0.008
6	2493	ENSG00000101306	MYLK2	7.82242	-1.0773	12.416	0.00043	0.011

**Table 4:** List of Inflammatory-Related DEGs in Nasopharyngeal Carcinoma based on the DAVID Database

No.	Entrez ID	Genes	Symbol	logFC	logFCPM	LR	P-value	FDR
1	2899	ENSG00000104321	TRPA1	5.9087	0.88547	8.8123	0,00299	0,041
2	2677	ENSG00000102468	HTR2A	76709	12.318	12.318	0,00045	0,012
3	6867	ENSG00000134259	NGF	8.2358	13.638	13.638	0,00022	0,009
4	5835	ENSG00000126583	PRKCG	5.8831	8.4911	8.4911	0,00357	0,046
5	9911	ENSG00000155897	ADCY8	9.2334	16.288	16.288	0,00005	0,008
6	12551	ENSG00000169248	CXCL9	5.8664	8.7434	8.7434	0,00311	0,042
7	12548	ENSG00000169245	CXCL10	5.834	8.829	8.829	0,00297	0,041
8	7723	ENSG00000138755	CXCL11	6.0372	9.291	9.291	0,00230	0,035

## Discussion

Based on pathway analysis using DAVID and PANTHER, there are 13 genes that play a role in the inflammatory response associated with Nasopharyngeal Carcinomafrom populations in Indonesia. The expression of the six genes from the PANTHER analysis based on several references, the *CXCL10 *gene overexpressed (upregulated) by binding to CXCR3 which promotes or induces the growth of breast cancer (Datta et al., 2006). COL20A1 occurs upregulated in glioblastoma and prostate cancer in response to pro-survival and inflammatory-related proliferation (Cai et al., 2018). High-expression *ACTC1* (upregulated) in the gene expression profiling (GEP) profiling of prostate cancer using microarrays (Huang et al., 2010). Increased Myosin light chain phosphorylation is affected by overregulation (upregulated) of the *MYLK* gene in PC12 in neuroendocrine tumor cells which causes damage to the apoptotic membrane (Mills et al., 1998). *ALOX15 *gene has decreased expression (downregulated) in colorectal tumorigenesis in colon cancer (Tian et al., 2017). The five genes showed the same level of expression in accordance with the reference obtained, while the *MYH2* gene itself was expressed in downregulated HNSCC (Head and neck squamous cell carcinoma) cells (Yang et al., 2019), different from the results obtained in Nasopharyngeal Carcinomasamples which actually shows the opposite.

From the results of the pathway analysis of Supplementary 3, the high expression of the *COL20A1 *gene in the inflammatory pathway mediated by cytokine and chemokine signaling shows the formation of a niche to mediate pro-inflammation by inducing an ECM (extracellular matrix) protein that triggers scaffolding and wound healing in cells or tissues, and causing destabilization or structural changes and the composition of the cell’s chemical matrix which are closely related to the development phase of a disease (Cataldi and Giacomo, 2018). Expression of the *MYLK* gene induces MLCK (Myosin Light Kinase) which activates the actin contraction with myosin so that the *MYH2* gene that influences myosin and *ACTC1* genes in high-expression actin. This triggers the occurrence of transcellular fissures that cause thinning of the cell shape, thinning of endothelial cells and breaking down glycocalyx. This causes inflammatory mediators to increase endothelial Ca^2+^ cytosol and cytokines IL-1β and TNF α initiate leukocyte migration, leukocytes activate endothelial and glycocalyx damaging agents such as free radical oxygen, and proteases (Herring and Paterson, 2018). Damage or weakening of the endothelium and very high oxygen content in cells and tissues triggers the formation of tumor microvessels (Kumar et al., 2009).

In this pathway the CXCL10 binds to the CXCR3 receptor in the signal transduction pathway that activates downstream G-protein Gβγ and Gαi classes, and includes the phosphoinositide 3-kinase (PI3K) pathway and mitogen-activated protein kinase (MAPK) which induces calcium uptake (Ca^2 +^) ) in cells, DNA synthesis, chemotaxis and cell proliferation (Ratman, 2010). The *CXCL10* gene expresses a cascade of MAPK and NF-kB synthesis, in which NF-kB plays an important role in triggering the production of chemokines that affect inflammatory cells in maintaining pro-inflammatory cancer, especially IL-10 from* CXCL10* gene expression (Shen et al ., 2006).

While the *ALOX15* gene affects the process of lipoxygenase synthesis in cells. The downregulated *ALOX15* gene plays a role in suppressing the signal of pro-inflammatory agents to suppress the inflammatory process. The enzymes from ALOX15 are easily induced and very high regulation in normal cells and are often found as inhibitors of pro-inflammatory signaling regulation through several mechanisms (Kuhn et al., 2002). Because of the downregulation of the *ALOX15* gene, the suppressor or inhibitor of inflammation decreases, causing the pro-inflammatory agent to activate in the chronic inflammatory process that triggers nasopharyngeal cancer.

The expression of the nine genes from the *DAVID *results, based on reference to the over-expression (upregulated) *CXCL9* gene, reduces the number of T immune cells in the organs and tumor microenvironment and reduces the secretion of IL-6 and TGF-β2 which triggers the development of prostate cancer (Tan et al., 2018). CLCX11 overexpression (upregulated) in colorectal cancer tissue and cell lines (CRC) (Gao et al., 2018). In the *TRPA1* gene overexpression (upregulated) in Nasopharyngeal Carcinomaand contribute to the development of lung cancer (Wu et al., 2016; Du et al., 2014). The genes in *HTR2A* have high expression correlated with high Gleason scores, as well as metastatic lymph nodes and bones in prostate cancer (Ballou et al., 2018). The *ADCY8* gene is upregulated in canine mammary malignancy in the expression of inflammation-related gene clusters in dogs (Pawlowski et al., 2013). *NGF* genes overexpress along with TrkA in pancreatic cancer tissue compared with normal pancreatic tissue, and increased NGF / TrkA expression is associated with invasion and pain caused by tumors (Zhu et al., 1999).

From the analysis of the Inflammatory Mediator Regulation of TRP Channels pathway in Supplementary 4, the high expression in the* TRPA1* gene associated with the pathway and reference shows an increase in intracellular Ca2 + concentration and an increase in cell survival in response to ROS H_2_O_2_, it shows that Ca^2 +^ influx mediated by TRPA1 can protect cancer cells against oxidative stress (Takahashi et al., 2018). Overexpression of the *NGF *gene in this pathway shows the role of pain caused by cancer growth during inflammation by stimulating TrkA which is a proto-oncogene that is also involved in PI3K activation related to proliferation and differentiation of cancer cells (Zhu et al., 1999).

Based on the Inflammatory Mediator Regulation of TRP Channels, ADCY8 plays a role in the activation of the cAMP and PKA chain reactions. ADCY8 plays a role through its binding with cAMP-activated protein kinase (PKA), which stimulates the initial and development of various tumors. From this process phosphorylates several target proteins, which lead to apoptosis, inhibit cell growth and stop cell movement (Caretta and Carla, 2011). The overexpressed *PRKCG* gene plays a role in the tumorigenesis of colon cancer, especially in the process of cancer migration. In the research of Dowling et al., (2018), analysis of* PRKCG* expression levels in colon cancer patients showed the regulation of these genes in cancerous tissue from 54% of patients examined. PKC also functions as the main receptor for phorbol esters, tumor promoter class (Griner and Kazanietz, 2007). In the *HTR2A* gene, the HTR2A receptor provides an inhibitory factor for alpha tumor necrosis (TNF-α) mediated by inflammation in primary aortic smooth muscle cells (Yu et al., 2009).

Based on the Inflammatory Mediator Regulation of TRP Channels Pathway the three genes namely *HTR2A*, *TRPA1* and *NGF* are connected to *TRPV1* and 2 other genes namely *ADCY8* and *PRKCG* refer to *TRPV4*. This is likely to trigger increased activity and sensitivity to the TRPV1 and TRVP4 channels in conditions of inflammation and hyperalgesia (Poole et al., 2013; Zhao et al., 2015). Phosphorylation by Protein Kinase C (PKC) and Protein Kinase A (PKA) phosphorylates and stimulates TRPV1 and TRPV4 channels with different locations in the development of heat hyperalgesia (Shuttleworth, 2009). The expression of NGF increases *TRPA1* expression and increased pain in people with hyperalgesia (Woolf et al., 1994). *PKC* and *PRKCG* redistribute in the expression of cytokines that mediate arachidonic acid that these genes are involved in the signaling pathway that induces arachidonic acid (Bordin et al., 2003). Arachidonic acid activates TRPV4 which requires protein phosphorylation mediated by PKA (Zheng et al., 2013). In sequence, TRPV4 mediates the induction of shear stress and endothelium which conditions increase ROS (Reactive Oxygen Species) (Bubolz et al., 2012). Based on the results and references in this pathway, it is closely related to the increase in ROS in Nasopharyngeal Carcinomacells to fight oxidative stress and the presence of inflammation-related hyperalgesia.

From the analysis of the Toll-Like Receptor Signaling Pathway in Supplementary 5, the presence of high expression in the* CXCL9, CXCL10*, and* CXCL 11* genes in this pathway is related to the JAK-STAT signaling pathway that produces chemokines that play a role in T cells. Chemokines in this pathway are *IP- 10 (CXCL10), MIG (CXCL9),* and *I-TAG (CXCL11)*. IFN γ activates Janus kinases (JAK) 1 and 2 which lead to phosphorylation of the STAT-1 protein, which then undergoes dimerization and binds to genes containing activated γ sequences including *CXCL9, CXCL10*, and *CXCL11* (Murray, 2007). The binding of *CXCL9, CXCL10, *and *CXCL11 to CXCR3* increases the amount of Ca2 + in intracellular (Cerny et al., 2015). *CXCR3* is highly expressed by activating Th1 and CD8 + lymphocyte cells and both cells recruit these cells into the inflammatory area. Based on this pathway IFNγ induces* CXCR3* ligands *(CXCL9, CXCL10, and CXCL11)* produced by endothelial cells, fibroblasts, mononuclear cells, and tumors. The CXC family of chemokines and their receptors, especially *CXCR3* with their ligands *CXCL9, CXCL10*, and *CXCL11* are involved in tumor and metastatic development through overexpression of CXCR3 in tumor cells which causes an excessive response to the expression of chemokines in tumors and inflammatory cells (Monteagudo, et al. , 2007). Based on these references *CXCL9, CXCL10* and *CXCL11 *are likely to influence tumorigenesis from inflammation-related nasopharyngeal cancer.
